# Is a large scale community programme as effective as a community rehabilitation programme delivered in the setting of a clinical trial?

**DOI:** 10.1186/1471-2288-13-103

**Published:** 2013-08-13

**Authors:** Elizabeth C Goyder, Mark Strong, Angela Green, Michael W Holmes, Gail Miles, Orla Reddington, Rod Lawson, Andrew Lee, Gurnam Basran

**Affiliations:** 1 ScHARR, University of Sheffield, Regent Court, 30 Regent St, Sheffield S1 4DA, UK; 2 NIHR CLAHRC South Yorkshire, NHS Rotherham CCG, Rotherham, UK; 3 Respiratory Medicine, Rotherham General Hospital, Rotherham, UK; 4 Sheffield Teaching Hospital NHS Trust, Sheffield, UK

**Keywords:** Pulmonary rehabilitation, Randomised controlled trials, Community programmes, Quality of life

## Abstract

**Background:**

The rationale for commissioning community pulmonary rehabilitation programmes is based on evidence from randomised clinical trials. However, there are a number of reasons why similar programmes might be less effective outside the environment of a clinical trial. These include a less highly selected patient group and less control over the fidelity of intervention delivery. The main objective of this study was therefore to test the hypothesis that the real-world programme would have similar outcomes to an intervention delivered in the context of a clinical trial.

**Methods:**

As part of the evaluation of an innovative community-based pulmonary rehabilitation programme (“BreathingSpace”), clinical and quality of life measures were collected before and after delivery of a rehabilitation programme. Baseline characteristics of participants and the change in symptoms and quality of life after the BreathingSpace programme were compared to measures collected in the community-based arm of a separate randomised trial of pulmonary rehabilitation.

**Results:**

Despite differences between the BreathingSpace participants and research participants in clinical status at baseline, patient reported symptoms and quality of life measures were similar. Improvements in both symptoms and quality of life were of the same order of magnitude despite the different contexts, setting and scale of the two intervention programmes. Whilst 73% (326/448) of those considered suitable for community rehabilitation in the trial and 80% (393/491) assessed as suitable for the BreathingSpace programme agreed to participate, less than half of participants completed rehabilitation, whether in a research or “real world” setting ( 47% and 45% respectively).

**Conclusion:**

The before-after changes in outcomes seen in a “real world” community rehabilitation programme are similar in magnitude to those seen in the intervention arm of a clinical trial. However suboptimal uptake and high dropout rates from rehabilitation amongst eligible participants occurs in both clinical trials and community based programmes and must be addressed if the benefits of rehabilitation for people with chronic lung disease are to be maximised.

## Background

The rationale for commissioning community pulmonary rehabilitation programmes is based on the results of randomised controlled trials (RCTs) in which rehabilitation has been shown to be effective and cost-effective in increasing exercise tolerance, reducing symptoms, improving quality of life and reducing hospital admissions [1-3].

Randomised controlled trials provide the “gold standard” for assessing effectiveness due to their high internal validity [4]. Only an RCT can properly allow for all confounders, both known and unknown, and a properly delivered RCT will minimise important sources of bias. This is in contrast to observational studies in which selection bias is often problematic, and in which it is very difficult to adjust for unknown confounders [5]. Guidance from the National Institute of Health and Clinical Excellence (NICE) and similar bodies on the effectiveness and cost-effectiveness of both drugs and complex interventions, including rehabilitation, is therefore based largely on the results of RCTs where these results are available [6].

However, it is not necessarily true that a randomised controlled trial has high external validity, in the sense that we cannot automatically expect to see the same effect size (or the same cost-effectiveness) when we deliver the intervention in a routine setting as was observed in the trial. There are a number of possible reasons for this: adherence to the intervention protocol may be less strict in the routine setting, and inclusion criteria for participants are likely to be more pragmatic. A trial typically attracts dedicated funding, whereas a programme that is delivered as part of usual health service provision must compete for scarce resources, particularly the time and commitment of clinicians and therapists. Lastly, the very nature of being observed in a trial can result in different behaviour by both patients and clinicians and therefore potentially a different outcome (the “Hawthorne effect”) [7].

In this study we compared the outcomes of pulmonary rehabilitation delivered in a randomised controlled clinical trial, with a similar programme delivered in a routine clinical setting. Our null hypothesis was that there was no difference in the change in outcome before and after the intervention in the clinical trial, compared with the before-after change in the routine clinical setting. A secondary aim was to evaluate uptake and dropout rates in both the BreathingSpace and randomised trial setting.

## Methods

We used a retrospective cohort study design and compared baseline characteristics and before-after change in outcomes between two groups: the community-based pulmonary rehabilitation intervention arm of the Waterhouse et al. randomised controlled trial that took place in the city of Sheffield, South Yorkshire, between 2004 and 2005 [8] and the group of patients who undertook pulmonary rehabilitation in the “BreathingSpace” community rehabilitation programme in the neighbouring town of Rotherham, South Yorkshire, between 2007 and 2008 [9]. Ethics approval for the BreathingSpace Evaluation and for the trial were granted by the Rotherham NHS Local Research Ethics Committee and South Sheffield NHS Local Research Ethics Committee respectively [8,9].

### Interventions

Both the trial and the BreathingSpace rehabilitation programmes were delivered in community venues and consisted of a total of 12 or 16 sessions delivered by support workers who were trained and supervised by experienced therapists. Classes were delivered over six weeks in the trial and over eight weeks in BreathingSpace. BreathingSpace had relatively broad inclusion criteria for participation, and included non-COPD patients, whilst the trial intervention was limited to patients with a specific diagnosis of COPD and MRC grade 3 (or worse) dyspnoea. Table 1 summarises the characteristics of the rehabilitation programmes delivered in the trial and in BreathingSpace and their inclusion criteria. More details are available [8,9]. Overall the main differences between the two programmes are those that might typically be found when comparing a randomised trial intervention and a community programme outside the context of a trial, in that although the trial was designed as a “pragmatic” trial and the trial protocol and report stresses that “access to the sessions was designed to follow usual clinical practice, reflecting ‘real life’ conditions” [8], the intervention did have more restrictive inclusion criteria (MRC severity grade of 3 or greater) and a more standardised intervention (“Programmes were identical in each venue, with exercises following a protocol and a core syllabus for each of the educational aspects” [8]).

**Table 1:** Intervention protocols for randomised trial and BreathingSpace

	**Randomised trial intervention protocol**	**BreathingSpace community rehabilitation programme**
Duration of programme	Twice weekly 2 hour classes for 6 weeks (total 12 hours)	Twice weekly 90 minute classes for 8 weeks (total 12 hours)
Delivery of programme	Delivered by physiotherapist and support worker, assessments performed by research team	Delivered by trained support workers supervised by physiotherapist or occupational therapist
Content of programme	1 hour of review, warm-up, exercise and cool-down; 1 hour for education; participants being encouraged to exercise between formal classes. Exercise diary kept between sessions and individualised exercise booklet provided at the end of the course.	1 hour of warm-up, exercise and cool-down; 30 minutes for education; participants being encouraged to exercise and keep an exercise diary between formal classes.
Inclusion/exclusion criteria	Diagnosis of COPD by respiratory physician, using GOLD guidelines	Diagnosed respiratory condition confirmed with spirometry;
	Medical Research Council (MRC) grade 3 or worse dyspnoea despite optimal medical care	Experiencing breathlessness in day to day life despite optimal respiratory medication
	Clinically stable at least 4 weeks before commencing rehabilitation.	No cardiac event in the past 3 months and any known cardiac condition well controlled and stable.

### Data collection and analysis

We obtained data for the intervention arm of the randomised controlled trial from the published trial report [8] and for BreathingSpace from an audit and evaluation project [9]. This included data on patient eligibility, recruitment and retention rates, baseline characteristics and post-rehabilitation follow-up data. Post-rehabilitation outcomes were measured immediately after completion of the rehabilitation programmes in both settings to maximise retention and minimise missing data at that point. Since the trial was restricted to patients with MRC breathlessness grades from 3–5 only and BreathingSpace recruited patients at all grades, to ensure comparability we restricted our primary analysis to patients from both programmes with MRC breathlessness grades from 3–5. We repeated the analyses including all patients to determine whether restricting the programme to more severe grades was likely to have an impact of overall effectiveness.

Whilst the trial cohort included all those who were randomised to the community rehabilitation programme and attended for follow up data collection, the BreathingSpace cohort included all those who had follow up data collected following the rehabilitation programme irrespective of their attendance for the programme, but excluding those who did not take up an offer of rehabilitation. We compared the following baseline characteristics: mean age, proportion male participants, mean body mass index, mean FEV1 (Forced expiratory volume in 1 second) mean FEV1 as a proportion of predicted, mean FVC (Forced Vital Capacity ie the volume of air that can forcibly be blown out after full inspiration), mean FVC as a proportion of predicted, mean relaxed vital capacity, mean FEV1/FVC and proportion in each MRC breathlessness grade [10]. We also compared baseline mean CRQ (Chronic Respiratory Disease Questionnaire) symptom severity [11], mean domain specific SF-36v2 (measuring quality of life defined in terms of physical functioning, role-physical, bodily pain, general health, vitality, social functioning, role-emotional, mental health, physical component summary, mental component summary), and overall SF-36v2 score (an summary measure of quality of life across all domains [12] and mean EQ-5D-3 L (a standardised measure of health status developed by the EuroQol Group which provides a simple, generic measure of health) [13]. These measures have all been widely used to evaluate self-reported outcomes of pulmonary rehabilitation [14] as they measure both symptoms in four domains (CRQ domains with range 1–7 for both individual and mean domain responses) and quality of life (domains of SF-36 with range 0–100; SF-6D and EQ-5D-3 L with range 0 (death) to 1 (perfect/full health)). Minimal clinically important differences for these outcome measures were defined, as for the randomised trial [8] as 0.5 points for CRQ domains, 5 points for SF-36 dimensions, 0.04 points for SF-6D score and 0.07 points for EQ-5D score.

We compared the following outcome measures, where outcomes were defined in terms of the difference in each measure before and after the rehabilitation intervention: CRQ symptoms (dyspnoea, fatigue, emotion, mastery), SF-36v2 domains, SF-36v2 overall score), and EQ-5D-3 L.

The primary objective was to evaluate whether the before/after mean difference was different between the two settings. We used the individual patient data from the community rehabilitation arm of the trial and the clinical outcomes data collected by the BreathingSpace programme patients to calculate mean before and after differences.

For baseline characteristics we tested for differences between the trial and BreathingSpace using unpaired t-tests for continuous measures, and a Chi-square test for measures expressed as proportions. For outcomes we tested for differences in the before-after differences using unpaired t-tests. We considered differences between the trial and BreathingSpace statistically significant if p < 0.05.

We also report the difference in mean before-after differences in outcomes between the trial community rehabilitation intervention arm and BreathingSpace, along with 95% confidence intervals.

## Results

### Recruitment and retention

In the clinical trial, out of 1041 patients assessed, 448 (43%) were considered suitable for the programme and 326 of those (73%) gave informed consent to randomisation. Of the 162 randomised to community re7habilitation, 111 (69%) attended the initial assessment of whom 76 had data at baseline and immediately post-rehabilitation. Subsequently, these participants attended about two-thirds of sessions (mean 62.53% (sd 7.3%); range 0-100%). Less than half (47%) of those randomised to community rehabilitation, who all had met eligibility criteria and given informed consent to participation, both attended and completed the programme.

In comparison, in BreathingSpace, out of the first 643 patients attending for assessment, 491 (76%) were assessed as suitable for rehabilitation and 393 (80%) of those agreed to attend. Audit data from the rehabilitation programme subsequently showed that only 45% (608/1355) of rehabilitation attendees attended more than 13 sessions and 37% (314/1355) attended less than eight sessions [15]. Only 451 rehabilitation participants provided symptom and quality of life data at baseline (Table 2) and the tables show the number of patients who provided data for specific outcome measures at both baseline (Tables 3 and 4) and post-rehabilitation (Tables 5 and 6). The different numbers of participants on which the results are based in Tables 2 to 6 (varying from 71 to 111 for the trial and from 451 to 308 for BreathingSpace) reflect differences in the completeness of data with respect to the different outcomes measured.

**Table 2:** Baseline clinical characteristics (at pre-rehabilitation assessment)

	**Randomised trial – community setting intervention group**					**BreathingSpace – all participants**					
	**n**	**Mean**	**SD**	**Min**	**Max**	**n**	**Mean**	**SD**	**Min**	**Max**	**P-value for difference between groups**
Age (years)	111	68.7	8.3	49	86	451	69.7	8.9	45	92	0.3
Body mass index (kg/m2)	111	25.4	5.6	14.0	44.0	421	27.5	6.5	12.5	66.2	0.001*
FEV1 (litres)	111	1.1	0.4	0.3	2.6	416	1.2	0.5	0.3	3.0	0.03*
Actual FEV1 as a proportion of predicted FEV1 (%)	111	45.1	16.3	16.8	89.8	417	48.3	16.6	15.0	117.0	0.07
FVC (litres)	111	2.7	0.9	1.2	5.3	415	2.4	0.8	0.9	6.5	0.002*
Actual FVC as a proportion of predicted FVC (%)	111	86.7	19.3	50.8	137.5	412	79.4	18.1	28.0	136.0	<0.001*
Relaxed vital capacity (litres)	111	2.7	0.9	1.4	5.1	398	2.5	0.8	0.7	6.1	0.04*
FEV1/FVC	111	0.4	0.1	0.2	1.0	412	0.5	0.1	0.2	0.9	<0.001*
	***n***	**%**				***n***	**%**				
***Gender***											
Female	49	44				219	49				0.5
Male	62	56				231	51				
*Total*	*111*	*100*				*450*	*100*				
**MRC breathlessness grade**											
1	-	-				8	2				<0.001*
2	-	-				68	16				
3	38	34				169	40				
4	37	33				132	32				
5	36	32				41	10				
*Total*	*111*	*100*				*418*	*100*				

**Table 3:** **Baseline self-reported symptoms and quality of life measures (at pre-rehabilitation assessment) including only participants MRC grade** ≥**3**

	**Randomised trial – community setting intervention group**			**BreathingSpace – participants MRC ≥3**			
	**n**	**Mean**	**SD**	**n**	**Mean**	**SD**	**P-value for difference in means**
**CRQ symptoms**							
Dyspnoea	75	3.2	0.9	329	2.9	1.0	0.002*
Fatigue	75	3.3	1.2	329	3.4	1.3	0.73
Emotion	75	4.4	1.3	329	4.4	1.4	0.92
Mastery	75	4.3	1.4	329	4.5	1.4	0.40
**SF-36v2 domains**							
Physical functioning	75	32.0	20.3	335	31.8	23.4	0.95
Role-physical	76	36.6	22.0	333	36.7	27.4	0.98
Bodily pain	74	58.2	27.9	313	54.4	29.7	0.30
General health	74	35.6	18.7	304	33.1	19.5	0.30
Vitality	75	38.7	18.6	306	37.7	21.2	0.68
Social functioning	76	49.7	30.8	313	52.6	33.0	0.47
Role-emotional	76	58.1	29.8	304	56.3	34.0	0.65
Mental health	76	65.1	21.5	310	66.4	20.5	0.63
PCS (Physical component summary)	71	32.2	7.4	291	31.2	9.7	0.32
MCS (Mental component summary)	71	42.3	12.2	291	43.7	13.1	0.38
**SF-6D**	71	0.61	0.11	291	0.59	0.12	0.10
**EQ-5D-3 L**	76	0.56	0.27	291	0.50	0.21	0.09

* significant at 5% level.

**Table 4:** Baseline self-reported symptoms and quality of life measures (at pre-rehabilitation assessment) – including BreathingSpace patients MRC grade < 3

	**Randomised trial – community setting intervention group**			**BreathingSpace – all participants**			
	**n**	**Mean**	**SD**	**n**	**Mean**	**SD**	**P-value for difference in means**
**CRQ symptoms**							
Dyspnoea	75	3.2	0.9	481	2.9	1.1	0.02*
Fatigue	75	3.3	1.2	481	3.5	1.3	0.2
Emotion	75	4.4	1.3	481	4.5	1.3	0.6
Mastery	75	4.3	1.4	481	4.5	1.4	0.2
**SF-36v2 domains**							
Physical functioning	75	32.0	20.3	443	32.0	23.2	1.0
Role-physical	76	36.6	22.0	442	36.7	26.7	1.0
Bodily pain	74	58.2	27.9	413	53.6	29.2	0.2
General health	74	35.6	18.7	401	33.4	20.0	0.4
Vitality	75	38.7	18.6	404	37.9	20.8	0.7
Social functioning	76	49.7	30.8	413	53.1	32.7	0.4
Role-emotional	76	58.1	29.8	404	55.6	34.1	0.5
Mental health	76	65.1	21.5	408	66.4	20.5	0.6
PCS (Physical component summary)	71	32.2	7.4	385	31.0	9.7	0.3
MCS (Mental component summary)	71	42.3	12.2	385	43.7	13.1	0.4
**SF-6D**	71	0.61	0.11	381	0.60	0.12	0.6
**EQ-5D-3 L**	76	0.56	0.27	385	0.53	0.23	0.4

**Table 5:** Impact of rehabilitation programme on respiratory symptoms and quality of life - including only participants MRC > =3

	**Randomised trial – community setting intervention group**			**BreathingSpace – participants MRC > =3Mean after-before difference**				
	**n**	**Mean before-after difference**	**SD**	**n**	**Mean before-after difference**	**SD**	**Difference in mean before-after differences (95% CI)**	**P-value for difference in mean before-after differences**
**CRQ symptoms**								
**Dyspnoea**	**74**	**0.29**	**1.18**	**329**	**0.55**	**1.20**	**0.26 (−0.04, 0.56)**	**0.09**
**Fatigue**	**75**	**0.58**	**1.12**	**329**	**0.51**	**1.21**	**−0.07 (−0.36, 0.21)**	**0.61**
**Emotion**	**74**	**0.39**	**0.98**	**329**	**0.34**	**1.15**	**−0.05 (−0.31, 0.21)**	**0.70**
**Mastery**	**75**	**0.36**	**1.08**	**329**	**0.27**	**1.24**	**−0.09 (−0.37, 0.19)**	**0.53**
**SF-36v2 domains**								
**Physical functioning**	**79**	**0.87**	**17.6**	**333**	**5.81**	**20.4**	**4.94 (0.36, 9.53)**	0.03*
**Role-physical**	**79**	**8.25**	**24.6**	**331**	**7.30**	**27.0**	**−0.95 (−7.27, 5.36)**	**0.77**
**Bodily pain**	**78**	**1.29**	**25.8**	**277**	**2.93**	**26.3**	**1.64 (−5.07, 8.35)**	**0.63**
**General health**	**77**	**3.93**	**15.7**	**263**	**3.17**	**17.0**	**−0.76 (−4.94, 3.42)**	**0.72**
**Vitality**	**79**	**8.15**	**16.8**	**269**	**7.59**	**21.4**	**−0.55 (−5.18, 4.08)**	**0.81**
**Social functioning**	**80**	**12.7**	**29.5**	**277**	**8.35**	**32.7**	**−4.31 (−12.04, 3.42)**	**0.27**
**Role-emotional**	**79**	**5.17**	**31.0**	**268**	**7.91**	**34.8**	**2.74 (−5.44, 10.93)**	**0.51**
**Mental health**	**80**	**5.81**	**15.7**	**274**	**4.76**	**19.5**	**−1.05 (−5.31, 3.20)**	**0.62**
**PCS (Physical component summary)**	**73**	**0.98**	**6.49**	**249**	**1.31**	**7.76**	**0.33 (−1.48, 2.13)**	**0.72**
**MCS (Mental component summary)**	**73**	**5.38**	**10.4**	**249**	**3.95**	**12.8**	**−1.43 (−4.35, 1.49)**	**0.34**
**SF-6D**	**72**	**0.014**	**0.10**	**245**	**0.030**	**0.11**	**0.016 (−0.010, 0.043)**	**0.23**
**EQ-5D-3 L**	**80**	**0.066**	**0.28**	**249**	**0.044**	**0.15**	**−0.022 (−0.086, 0.042)**	**0.51**

* significant at 5% level.

**Table 6:** Impact of rehabilitation programme on respiratory symptoms and quality of life– including BreathingSpace patients MRC grade 1-2

	**Randomised trial – community setting intervention group**			**BreathingSpace – all participants**				
	**n**	**Mean before-after difference**	**SD**	**n**	**Mean before-after difference**	**SD**	**Difference in mean before-after differences (95% CI)**	**P-value for difference in mean before-after differences**
**CRQ symptoms**								
**Dyspnoea**	**74**	**0.29**	**1.18**	**481**	**0.61**	**1.25**	**0.32 (0.03, 0.62)**	0.03*
**Fatigue**	**75**	**0.58**	**1.12**	**481**	**0.48**	**1.22**	**−0.10 (−0.38, 0.18)**	**0.31**
**Emotion**	**74**	**0.39**	**0.98**	**481**	**0.36**	**1.15**	**−0.03 (−0.27, 0.22)**	**0.79**
**Mastery**	**75**	**0.36**	**1.08**	**481**	**0.32**	**1.20**	**−0.04 (−0.31, 0.23)**	**0.86**
**SF-36v2 domains**								
**Physical functioning**	**79**	**0.87**	**17.6**	**438**	**5.47**	**20.1**	**4.61 (0.16, 9.06)**	**0.08**
**Role-physical**	**79**	**8.25**	**24.6**	**437**	**7.36**	**26.6**	**−0.9 (−7.04, 5.24)**	**0.57**
**Bodily pain**	**78**	**1.29**	**25.8**	**356**	**2.55**	**26.3**	**1.25 (−5.30, 7.80)**	**0.71**
**General health**	**77**	**3.93**	**15.7**	**337**	**3.29**	**16.5**	**−0.64 (−4.68, 3.40)**	**0.78**
**Vitality**	**79**	**8.15**	**16.8**	**345**	**6.75**	**20.5**	**−1.4 (−5.82, 3.03)**	**0.42**
**Social functioning**	**80**	**12.7**	**29.5**	**356**	**7.97**	**31.7**	**−4.69 (−12.16, 2.79)**	**0.11**
**Role-emotional**	**79**	**5.17**	**31.0**	**346**	**7.77**	**32.9**	**2.6 (−5.26, 10.46)**	**0.38**
**Mental health**	**80**	**5.81**	**15.7**	**351**	**4.65**	**18.7**	**−1.16 (−5.23, 2.92)**	**0.48**
**PCS (Physical component summary)**	**73**	**0.98**	**6.49**	**317**	**1.11**	**7.5**	**0.13 (−1.61, 1.87)**	**0.84**
**MCS (Mental component summary)**	**73**	**5.38**	**10.4**	**317**	**3.78**	**12.1**	**−1.59 (−4.39, 1.20)**	**0.24**
**SF-6D**	**72**	**0.014**	**0.10**	**308**	**0.029**	**0.11**	**0.015 (−0.011, 0.040)**	**0.30**
**EQ-5D-3 L**	**80**	**0.066**	**0.28**	**317**	**0.039**	**0.14**	**−0.027 (−0.090, 0.036)**	**0.42**

* significant at 5% level.

### Baseline characteristics

There were no significant differences in either the mean age or the gender distribution between the trial intervention arm and BreathingSpace (Table 2). The community participants had higher body mass indices but had significantly better lung function. This is consistent with the smaller proportion at MRC Grade 5 in the BreathingSpace (10% versus 32%) and is due at least in part to the different inclusion criteria of the trial and BreathingSpace. BreathingSpace accepts patients with MRC grades 1 and 2 breathlessness, whereas these participants were excluded from the trial. Despite these differences, patient reported symptoms and quality of life measures were similar in the two populations (Tables 3 and 4).

### Respiratory symptoms and quality of life outcomes

Pre-rehabilitation and post-rehabilitation outcomes were collected for 329 BreathingSpace participants and 80 trial participants (Tables 5 and 6). Both groups improved with similar changes seen in the trial and BreathingSpace. The differences in mean before-after differences in outcomes between the trial community rehabilitation intervention arm and BreathingSpace were all small, and smaller than the clinically important difference. Confidence intervals for the differences in differences contained zero (the null value) for all outcomes except the Physical Functioning domain, where participants in BreathingSpace improved more than those in the trial (Table 5). However, this finding should be viewed with caution given that the overall Physical component summary improvement and overall SF-36 improvements were similar between groups. Repeating the analyses including 152 Breathing Space patients with MRC Grades 1–2 (a total of 481 patients) in the Breathing Space cohort gave similar results (Table 6).

## Discussion

The primary objective was to evaluate whether the before/after mean differences in outcomes seen at BreathingSpace were different to those reported in the intervention arm of the trial. Despite some differences between BreathingSpace participants and research participants in clinical status at baseline, patient reported symptoms and quality of life measures were similar in the two populations. Improvements in both symptoms and quality of life were of the same order of magnitude in both programmes despite the different contexts, setting and scale of the two intervention programmes. In particular, the inclusion of patients with less clinically severe disease (less than MRC Grade 3) does not appear to have reduced the overall effectiveness, a finding consistent with previous evaluations [16]. In both the clinical trial and the larger scale programme, a high proportion of those originally assessed, and who might have potentially benefited, either did not start or did not complete the programme. This suggests that the barriers to participation and completion of rehabilitation are not only due to the additional constraints of involvement in a trial but also occur in “real world” non-research settings. There is no clear consensus or evidence base to support any particular threshold for regarding attendance as adequate before an individual can be said to have received an adequate “dose” of rehabilitation in this context. The relationship between attendance and outcomes will be confounded by other factors that will influence both attendance and outcomes, particularly changes in health status. We suspect that poor attendance could be causally associated with both poorer health (participants not well enough to attend) and with better health (participants feeling well enough to take up other normal activities and responsibilities and therefore choosing not to attend) so interpreting whether (and why) better attendance might predict better or worse outcomes requires further research.

This analysis is based on a comparison of a group of patients recruited to a clinical trial of community-based pulmonary rehabilitation and a group of patients receiving community-based pulmonary rehabilitation in clinical practice. The first group were exposed to pulmonary rehabilitation as part of a Sheffield based randomised trial, funded by the Health Technology Appraisal Programme (NIHR HTA programme). The second group were exposed to rehabilitation as part of a large community programme based in the purpose-built “BreathingSpace” facility in the neighbouring town of Rotherham. The content and delivery of both programmes was based on a similar interpretation of the evidence base for COPD rehabilitation programmes at that time [1]. This provides a unique opportunity to compare the impact of rehabilitation seen in a controlled trial with the impact achieved in a routine community setting. However, whilst we have assumed that as both groups were identified from geographically adjacent populations and both identified as suitable for community-based pulmonary rehabilitation, they represent similar populations, there is always the potential for patients included in research studies being selected to be different from the patients that take up the intervention in clinical practice.

The other main weaknesses of this analysis are those associated with any secondary analysis, in that the data available for analysis are limited to those that were collected for the original analyses. Potentially useful missing information included measures of baseline differences such as the presence or absence of co-morbidities, smoking status and information on other relevant clinical measures such as walking distance and health care service use. However from the data that were collected, we are reasonably confident that we are comparing similar populations who received similar programmes.

Some studies that have compared the results of randomised trials and observational studies have found that they generate similar results [17]. Although a number of studies have considered the rationale for expecting differences between trials and observational studies, none to date have considered the empirical evidence for a difference in the clinical effectiveness of a specific complex intervention such as pulmonary rehabilitation, when replicated in a non-research setting.

Other authors have shown that the effect sizes observed in randomised trials can be quite different to those observed in non-randomised studies of the same intervention [18]. This is not surprising given the problems of internal validity (i.e. bias and confounding) that are inherent in non-randomised studies. Our question is somewhat different and relates to the external validity of a randomised trial. Can we expect to see the same outcomes in the routine clinical setting that we saw in the trial? Based on the results we present for pulmonary rehabilitation we conclude that, outcomes similar to those achieved in clinical trials can be achieved in the “real world”.

## Conclusions

We conclude that the before-after changes in outcomes seen in a “real world” community rehabilitation programme are similar in magnitude to those seen in the intervention arm of a clinical trial. However, the relatively low participation and completion rates that are observed in the context of trials, and which might be thought to be specific to the trial setting (due for example to the requirements of an onerous consent process) also exist in “real world” programmes. The barriers to participation in *any* pulmonary rehabilitation programme, trial or otherwise, that are indicated by these findings need to be addressed if the potential benefits of rehabilitation for a larger number of people with chronic lung disease are to be maximised.

## Competing interests

AG, GS, OR and GB were involved in the delivery of BreathingSpace programmes. RL was principal investigator for the NIHR-funded pulmonary rehabilitation trial and has received payment for Board membership (Novartis, GSK) and received grants and payment or expenses for presentations and meetings (GSK, Novartis, Boehringer, Nycomed, Astra Zeneca).

## Authors’ contributions

EG, MS, AG and GB conceived the study, and participated in its design and coordination. MS and MH carried out the statistical analyses and EG wrote the first draft of the manuscript. RL, OR, GB and GM also provided information about the interventions and data collection process in both settings. All authors contributed to the interpretation of the findings and to drafting the manuscript. All authors read and approved the final manuscript.

## Pre-publication history

The pre-publication history for this paper can be accessed here:

http://www.biomedcentral.com/1471-2288/13/103/prepub
